# Dimethyl 2,2′-di­nitro­biphenyl-4,4′-di­carboxyl­ate

**DOI:** 10.1107/S1600536814003067

**Published:** 2014-02-15

**Authors:** Ryan L. Lehane, James A. Golen, Arnold L. Rheingold, David R. Manke

**Affiliations:** a Department of Chemistry and Biochemistry, University of Massachusetts Dartmouth, 285 Old Westport Road, North Dartmouth, MA 02747, USA; b Department of Chemistry, University of California, San Diego, 9500 Gilman Drive, La Jolla, CA 92093, USA

## Abstract

The title compound, C_16_H_12_N_2_O_8_, exhibits two near-planar aromatic ester groups with ar­yl–ester dihedral angles of 2.1 (2) and 4.2 (3)°. The dihedral angle between the aromatic rings is 58.0 (1)°. The two nitro groups are tilted slightly from the plane of the aromatic rings, making dihedral angles of 14.1 (1) and 8.2 (2)°. In the crystal, mol­ecules are connected by weak C—H⋯O inter­actions, forming a three-dimensional network.

## Related literature   

For the synthesis of the title compound, see: Ol’khovik *et al.* (2008 ▶). For the structure of 2,2′-di­nitro­biphenyl-4,4′-di­carb­oxy­lic acid, see: Wu *et al.* (2010 ▶). For metal-organic frameworks and coordination polymers featuring this linker, see: Qu (2007 ▶); Li, Zhou *et al.* (2011 ▶); Li, Li *et al.* (2011 ▶); Li, Zhang *et al.* (2011 ▶); Zhang, Ma *et al.* (2011 ▶); Zhang, Jing *et al.* (2011 ▶); Li, Yang *et al.* (2012 ▶); Zhang, Li *et al.* (2012 ▶).
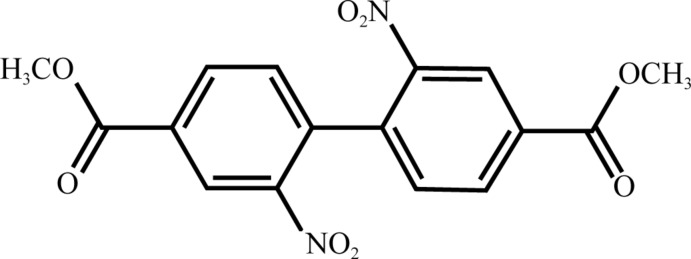


## Experimental   

### 

#### Crystal data  

C_16_H_12_N_2_O_8_
*M* *_r_* = 360.28
Triclinic, 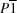

*a* = 8.0520 (5) Å
*b* = 10.4193 (7) Å
*c* = 10.5310 (11) Å
α = 108.423 (4)°
β = 95.142 (4)°
γ = 111.617 (3)°
*V* = 757.71 (11) Å^3^
*Z* = 2
Mo *K*α radiation
μ = 0.13 mm^−1^
*T* = 90 K
0.28 × 0.20 × 0.15 mm

#### Data collection  

Bruker APEXII CCD diffractometer
Absorption correction: multi-scan (*SADABS*; Bruker, 2005 ▶) *T*_min_ = 0.965, *T*_max_ = 0.981
9906 measured reflections
2787 independent reflections
2297 reflections with *I* > 2σ(*I*)
*R*_int_ = 0.026

#### Refinement  

*R*[*F* ^2^ > 2σ(*F* ^2^)] = 0.034
*wR*(*F* ^2^) = 0.096
*S* = 1.02
2787 reflections
237 parameters
H-atom parameters constrained
Δρ_max_ = 0.21 e Å^−3^
Δρ_min_ = −0.20 e Å^−3^

### 

Data collection: *APEX2* (Bruker, 2005 ▶); cell refinement: *SAINT* (Bruker, 2005 ▶); data reduction: *SAINT*; program(s) used to solve structure: *SHELXS97* (Sheldrick, 2008 ▶); program(s) used to refine structure: *SHELXL97* (Sheldrick, 2008 ▶); molecular graphics: *SHELXTL* (Sheldrick, 2008 ▶); software used to prepare material for publication: *SHELXTL*.

## Supplementary Material

Crystal structure: contains datablock(s) I, New_Global_Publ_Block. DOI: 10.1107/S1600536814003067/ff2126sup1.cif

Structure factors: contains datablock(s) I. DOI: 10.1107/S1600536814003067/ff2126Isup2.hkl

Supporting information file. DOI: 10.1107/S1600536814003067/ff2126Isup3.cml

CCDC reference: 986204

Additional supporting information:  crystallographic information; 3D view; checkCIF report

## Figures and Tables

**Table 1:** Hydrogen-bond geometry (Å, °)

*D*—H⋯*A*	*D*—H	H⋯*A*	*D*⋯*A*	*D*—H⋯*A*
C16—H16*A*⋯O3^i^	0.98	2.50	3.253 (2)	134
C1—H1*C*⋯O2^ii^	0.98	2.57	3.5214 (19)	164
C14—H14*A*⋯O2^iii^	0.95	2.47	3.2399 (19)	138
C8—H8*A*⋯O3^iv^	0.95	2.41	3.3239 (19)	162

Symmetry codes: (i) 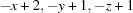; (ii) 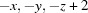; (iii) 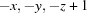; (iv) 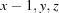.

**Fig. 1.**
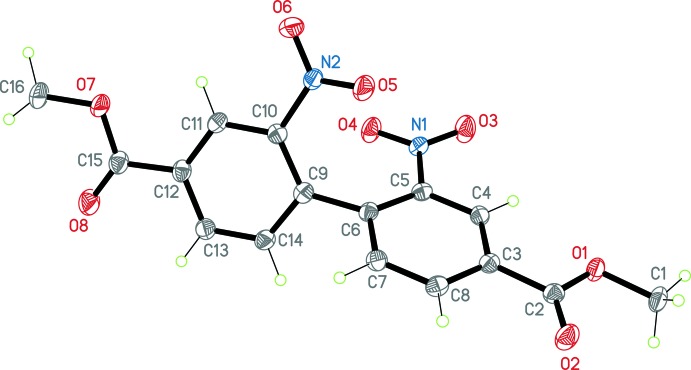
Molecular structure of the title compound, showing the atom labelling scheme. Displacement ellipsoids are drawn at the 50% probability level. H atoms are presented as spheres of arbitrary radius.

**Fig. 2.**
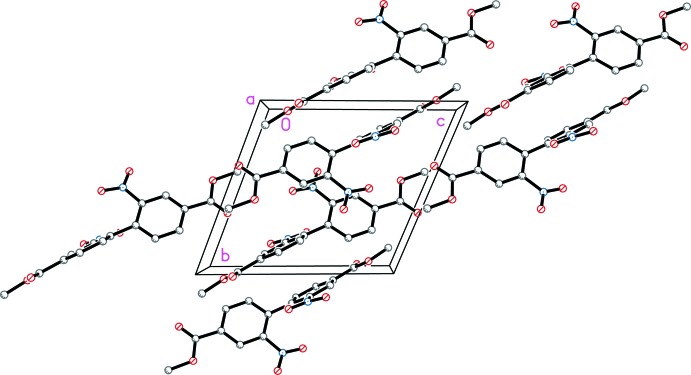
Molecular packing of the title compound.

## supplementary crystallographic information

### 1. Comment

Biphenyl-4,4'-dicarboxylate and its derivatives are widely used in metal-organic frameworks (MOFs) as linkers. One of the many advantages of MOFs is the ability to incorporate different functional groups within their pores. As a part of our efforts in this field, we prepared the previously reported dimethyl 2,2'-dinitrobiphenyl-4,4'-dicarboxylate (Ol'khovik *et al.* 2008) and report its structure herein.

The molecular structure of the title compound is shown in Figure 1. The structure demonstrates two near planar aromatic ester groups with aryl-ester dihedral angles of 2.1 (2)° and 4.2 (3)°. The two aromatic rings demonstrate a dihedral angle of 58.0 (1)°. The nitro groups are skewed slightly with aryl-nitro dihedral angles of 8.2 (2)° and 14.1 (1)°. No π-π interactions were noted between the aromatic rings. The packing for the title compound is shown in Figure 2.

### 2. Experimental

The compound was prepared by literature procedure (Ol'khovik *et al.* 2008). Crystals suitable for single-crystal X-ray analysis were grown by slow evaporation of an ethanol solution.

### 3. Refinement

All non-hydrogen atoms refined anisotropically by full matrix least squares on F^2^. All hydrogen atoms were placed in calculated positions and then refined with riding model with C—H lengths of 0.95 Å for (CH) and 0.98 Å for (CH_3_) and with isotropic displacement parameters set to 1.20 and 1.50 times *U*_eq_ of the parent C atom.

### Crystal data

**Table:**

C_16_H_12_N_2_O_8_	*Z* = 2
*M**_r_* = 360.28	*F*(000) = 372
Triclinic, *P*1	*D*_x_ = 1.579 Mg m^−^^3^
Hall symbol: -P 1	Mo *K*α radiation, λ = 0.71073 Å
*a* = 8.0520 (5) Å	Cell parameters from 5153 reflections
*b* = 10.4193 (7) Å	θ = 2.8–25.4°
*c* = 10.5310 (11) Å	µ = 0.13 mm^−^^1^
α = 108.423 (4)°	*T* = 90 K
β = 95.142 (4)°	BLOCK, yellow
γ = 111.617 (3)°	0.28 × 0.20 × 0.15 mm
*V* = 757.71 (11) Å^3^	

### Data collection

**Table:**

Bruker APEXII CCD diffractometer	2787 independent reflections
Radiation source: fine-focus sealed tube	2297 reflections with *I* > 2σ(*I*)
Graphite monochromator	*R*_int_ = 0.026
φ and ω scans	θ_max_ = 25.4°, θ_min_ = 2.8°
Absorption correction: multi-scan (*SADABS*; Bruker, 2005)	*h* = −9→9
*T*_min_ = 0.965, *T*_max_ = 0.981	*k* = −12→12
9906 measured reflections	*l* = −12→12

### Refinement

**Table:**

Refinement on *F*^2^	Primary atom site location: structure-invariant direct methods
Least-squares matrix: full	Secondary atom site location: difference Fourier map
*R*[*F*^2^ > 2σ(*F*^2^)] = 0.034	Hydrogen site location: inferred from neighbouring sites
*wR*(*F*^2^) = 0.096	H-atom parameters constrained
*S* = 1.02	*w* = 1/[σ^2^(*F*_o_^2^) + (0.0515*P*)^2^ + 0.234*P*] where *P* = (*F*_o_^2^ + 2*F*_c_^2^)/3
2787 reflections	(Δ/σ)_max_ = 0.001
237 parameters	Δρ_max_ = 0.21 e Å^−^^3^
0 restraints	Δρ_min_ = −0.20 e Å^−^^3^

### Special details

**Table:**

Geometry. All e.s.d.'s (except the e.s.d. in the dihedral angle between two l.s. planes) are estimated using the full covariance matrix. The cell e.s.d.'s are taken into account individually in the estimation of e.s.d.'s in distances, angles and torsion angles; correlations between e.s.d.'s in cell parameters are only used when they are defined by crystal symmetry. An approximate (isotropic) treatment of cell e.s.d.'s is used for estimating e.s.d.'s involving l.s. planes.
Refinement. Refinement of *F*^2^ against ALL reflections. The weighted *R*-factor *wR* and goodness of fit *S* are based on *F*^2^, conventional *R*-factors *R* are based on *F*, with *F* set to zero for negative *F*^2^. The threshold expression of *F*^2^ > σ(*F*^2^) is used only for calculating *R*-factors(gt) *etc*. and is not relevant to the choice of reflections for refinement. *R*-factors based on *F*^2^ are statistically about twice as large as those based on *F*, and *R*- factors based on ALL data will be even larger.

### Fractional atomic coordinates and isotropic or equivalent isotropic displacement parameters (Å^2^)

**Table:**

	*x*	*y*	*z*	*U*_iso_*/*U*_eq_	
O1	0.28379 (14)	−0.04398 (11)	0.86338 (10)	0.0223 (3)	
O2	0.02811 (15)	−0.01405 (13)	0.81152 (11)	0.0283 (3)	
O3	0.80395 (15)	0.15657 (14)	0.70622 (13)	0.0350 (3)	
O4	0.80373 (15)	0.23016 (13)	0.53753 (11)	0.0283 (3)	
O5	0.72326 (16)	0.49239 (12)	0.69694 (11)	0.0327 (3)	
O6	0.93059 (15)	0.61445 (13)	0.61157 (11)	0.0323 (3)	
O7	0.80462 (15)	0.55730 (12)	0.14341 (11)	0.0292 (3)	
O8	0.56913 (17)	0.35679 (13)	−0.01199 (11)	0.0355 (3)	
N1	0.72907 (16)	0.18850 (13)	0.62186 (13)	0.0196 (3)	
N2	0.78359 (17)	0.51402 (14)	0.59936 (12)	0.0206 (3)	
C1	0.1936 (2)	−0.12748 (18)	0.94307 (16)	0.0253 (4)	
H1A	0.2829	−0.1467	0.9949	0.038*	
H1B	0.0943	−0.2224	0.8810	0.038*	
H1C	0.1427	−0.0698	1.0072	0.038*	
C2	0.1814 (2)	0.00368 (16)	0.79992 (15)	0.0199 (3)	
C3	0.2780 (2)	0.07985 (15)	0.71294 (14)	0.0181 (3)	
C4	0.4574 (2)	0.10299 (15)	0.70668 (14)	0.0182 (3)	
H4A	0.5234	0.0717	0.7606	0.022*	
C5	0.53980 (19)	0.17125 (15)	0.62247 (15)	0.0179 (3)	
C6	0.4489 (2)	0.21904 (15)	0.53960 (15)	0.0181 (3)	
C7	0.2683 (2)	0.19281 (16)	0.54750 (15)	0.0199 (3)	
H7A	0.2004	0.2214	0.4919	0.024*	
C8	0.1845 (2)	0.12668 (16)	0.63336 (15)	0.0201 (3)	
H8A	0.0622	0.1132	0.6379	0.024*	
C9	0.5175 (2)	0.28358 (16)	0.43647 (15)	0.0186 (3)	
C10	0.6699 (2)	0.41620 (16)	0.45972 (15)	0.0187 (3)	
C11	0.7209 (2)	0.46404 (16)	0.35496 (15)	0.0196 (3)	
H11A	0.8284	0.5524	0.3740	0.024*	
C12	0.6138 (2)	0.38191 (16)	0.22211 (15)	0.0208 (3)	
C13	0.4585 (2)	0.25263 (17)	0.19660 (16)	0.0232 (3)	
H13A	0.3833	0.1967	0.1060	0.028*	
C14	0.4123 (2)	0.20443 (16)	0.30184 (15)	0.0217 (3)	
H14A	0.3060	0.1149	0.2819	0.026*	
C15	0.6575 (2)	0.42811 (17)	0.10439 (16)	0.0239 (4)	
C16	0.8490 (3)	0.6119 (2)	0.03466 (17)	0.0354 (4)	
H16A	0.9564	0.7083	0.0729	0.053*	
H16B	0.7443	0.6240	−0.0062	0.053*	
H16C	0.8765	0.5404	−0.0362	0.053*	

### Atomic displacement parameters (Å^2^)

**Table:**

	*U*^11^	*U*^22^	*U*^33^	*U*^12^	*U*^13^	*U*^23^
O1	0.0235 (6)	0.0248 (6)	0.0241 (6)	0.0100 (5)	0.0094 (4)	0.0154 (5)
O2	0.0227 (6)	0.0362 (7)	0.0344 (7)	0.0134 (5)	0.0140 (5)	0.0205 (5)
O3	0.0253 (6)	0.0526 (8)	0.0442 (7)	0.0200 (6)	0.0117 (5)	0.0348 (6)
O4	0.0251 (6)	0.0383 (7)	0.0328 (6)	0.0153 (5)	0.0158 (5)	0.0230 (6)
O5	0.0419 (7)	0.0275 (6)	0.0197 (6)	0.0035 (5)	0.0098 (5)	0.0099 (5)
O6	0.0255 (6)	0.0306 (6)	0.0274 (6)	−0.0018 (5)	0.0033 (5)	0.0109 (5)
O7	0.0336 (7)	0.0274 (6)	0.0213 (6)	0.0032 (5)	0.0068 (5)	0.0140 (5)
O8	0.0512 (8)	0.0268 (6)	0.0189 (6)	0.0071 (6)	0.0035 (5)	0.0088 (5)
N1	0.0185 (7)	0.0177 (6)	0.0228 (7)	0.0066 (5)	0.0056 (5)	0.0087 (5)
N2	0.0227 (7)	0.0186 (6)	0.0208 (7)	0.0072 (6)	0.0059 (5)	0.0095 (5)
C1	0.0312 (9)	0.0242 (8)	0.0245 (8)	0.0097 (7)	0.0113 (7)	0.0149 (7)
C2	0.0200 (8)	0.0174 (7)	0.0190 (8)	0.0060 (6)	0.0042 (6)	0.0050 (6)
C3	0.0196 (8)	0.0153 (7)	0.0173 (7)	0.0064 (6)	0.0051 (6)	0.0042 (6)
C4	0.0207 (8)	0.0158 (7)	0.0170 (7)	0.0074 (6)	0.0029 (6)	0.0057 (6)
C5	0.0168 (7)	0.0160 (7)	0.0194 (7)	0.0065 (6)	0.0050 (6)	0.0049 (6)
C6	0.0213 (8)	0.0130 (7)	0.0173 (7)	0.0054 (6)	0.0044 (6)	0.0045 (6)
C7	0.0195 (8)	0.0194 (8)	0.0228 (8)	0.0094 (6)	0.0041 (6)	0.0090 (6)
C8	0.0184 (8)	0.0191 (8)	0.0223 (8)	0.0080 (6)	0.0066 (6)	0.0065 (6)
C9	0.0198 (8)	0.0174 (7)	0.0223 (8)	0.0106 (6)	0.0069 (6)	0.0083 (6)
C10	0.0200 (8)	0.0180 (7)	0.0183 (8)	0.0086 (6)	0.0042 (6)	0.0062 (6)
C11	0.0210 (8)	0.0160 (7)	0.0233 (8)	0.0078 (6)	0.0069 (6)	0.0087 (6)
C12	0.0263 (8)	0.0187 (8)	0.0203 (8)	0.0113 (7)	0.0069 (6)	0.0083 (6)
C13	0.0272 (8)	0.0191 (8)	0.0203 (8)	0.0074 (7)	0.0021 (6)	0.0073 (6)
C14	0.0218 (8)	0.0173 (7)	0.0248 (8)	0.0068 (6)	0.0040 (6)	0.0087 (6)
C15	0.0309 (9)	0.0197 (8)	0.0223 (9)	0.0111 (7)	0.0069 (7)	0.0089 (7)
C16	0.0409 (10)	0.0378 (10)	0.0272 (9)	0.0071 (8)	0.0099 (8)	0.0225 (8)

### Geometric parameters (Å, º)

**Table:**

O1—C2	1.3363 (18)	C5—C6	1.405 (2)
O1—C1	1.4469 (17)	C6—C7	1.393 (2)
O2—C2	1.2024 (18)	C6—C9	1.494 (2)
O3—N1	1.2256 (16)	C7—C8	1.381 (2)
O4—N1	1.2188 (16)	C7—H7A	0.9500
O5—N2	1.2239 (16)	C8—H8A	0.9500
O6—N2	1.2197 (16)	C9—C14	1.392 (2)
O7—C15	1.3315 (19)	C9—C10	1.399 (2)
O7—C16	1.4486 (18)	C10—C11	1.383 (2)
O8—C15	1.1995 (19)	C11—C12	1.384 (2)
N1—C5	1.4682 (18)	C11—H11A	0.9500
N2—C10	1.4708 (19)	C12—C13	1.385 (2)
C1—H1A	0.9800	C12—C15	1.491 (2)
C1—H1B	0.9800	C13—C14	1.381 (2)
C1—H1C	0.9800	C13—H13A	0.9500
C2—C3	1.485 (2)	C14—H14A	0.9500
C3—C8	1.387 (2)	C16—H16A	0.9800
C3—C4	1.385 (2)	C16—H16B	0.9800
C4—C5	1.374 (2)	C16—H16C	0.9800
C4—H4A	0.9500		
			
C2—O1—C1	115.18 (11)	C6—C7—H7A	118.9
C15—O7—C16	115.00 (12)	C3—C8—C7	120.31 (14)
O4—N1—O3	122.78 (12)	C3—C8—H8A	119.8
O4—N1—C5	119.80 (12)	C7—C8—H8A	119.8
O3—N1—C5	117.40 (12)	C14—C9—C10	116.34 (13)
O6—N2—O5	123.65 (13)	C14—C9—C6	115.67 (13)
O6—N2—C10	118.19 (12)	C10—C9—C6	127.94 (13)
O5—N2—C10	118.13 (12)	C11—C10—C9	122.64 (13)
O1—C1—H1A	109.5	C11—C10—N2	116.29 (13)
O1—C1—H1B	109.5	C9—C10—N2	121.07 (13)
H1A—C1—H1B	109.5	C12—C11—C10	119.37 (14)
O1—C1—H1C	109.5	C12—C11—H11A	120.3
H1A—C1—H1C	109.5	C10—C11—H11A	120.3
H1B—C1—H1C	109.5	C11—C12—C13	119.30 (14)
O2—C2—O1	123.67 (14)	C11—C12—C15	122.49 (14)
O2—C2—C3	124.01 (14)	C13—C12—C15	118.20 (14)
O1—C2—C3	112.32 (12)	C14—C13—C12	120.55 (14)
C8—C3—C4	118.93 (13)	C14—C13—H13A	119.7
C8—C3—C2	118.95 (13)	C12—C13—H13A	119.7
C4—C3—C2	122.11 (13)	C13—C14—C9	121.72 (14)
C5—C4—C3	120.01 (14)	C13—C14—H14A	119.1
C5—C4—H4A	120.0	C9—C14—H14A	119.1
C3—C4—H4A	120.0	O8—C15—O7	124.10 (14)
C4—C5—C6	122.65 (13)	O8—C15—C12	123.56 (14)
C4—C5—N1	115.70 (13)	O7—C15—C12	112.33 (13)
C6—C5—N1	121.64 (13)	O7—C16—H16A	109.5
C7—C6—C5	115.81 (13)	O7—C16—H16B	109.5
C7—C6—C9	116.20 (13)	H16A—C16—H16B	109.5
C5—C6—C9	127.81 (13)	O7—C16—H16C	109.5
C8—C7—C6	122.27 (14)	H16A—C16—H16C	109.5
C8—C7—H7A	118.9	H16B—C16—H16C	109.5
			
C1—O1—C2—O2	3.3 (2)	C7—C6—C9—C10	122.53 (16)
C1—O1—C2—C3	−176.29 (11)	C5—C6—C9—C10	−62.6 (2)
O2—C2—C3—C8	−3.9 (2)	C14—C9—C10—C11	−3.2 (2)
O1—C2—C3—C8	175.74 (12)	C6—C9—C10—C11	179.48 (14)
O2—C2—C3—C4	177.44 (14)	C14—C9—C10—N2	175.83 (13)
O1—C2—C3—C4	−2.96 (19)	C6—C9—C10—N2	−1.5 (2)
C8—C3—C4—C5	−0.1 (2)	O6—N2—C10—C11	−12.78 (19)
C2—C3—C4—C5	178.64 (13)	O5—N2—C10—C11	165.42 (13)
C3—C4—C5—C6	−0.5 (2)	O6—N2—C10—C9	168.14 (13)
C3—C4—C5—N1	−179.15 (12)	O5—N2—C10—C9	−13.7 (2)
O4—N1—C5—C4	170.63 (13)	C9—C10—C11—C12	2.8 (2)
O3—N1—C5—C4	−7.86 (19)	N2—C10—C11—C12	−176.24 (13)
O4—N1—C5—C6	−8.1 (2)	C10—C11—C12—C13	−0.6 (2)
O3—N1—C5—C6	173.45 (13)	C10—C11—C12—C15	178.61 (13)
C4—C5—C6—C7	−0.1 (2)	C11—C12—C13—C14	−1.1 (2)
N1—C5—C6—C7	178.50 (12)	C15—C12—C13—C14	179.67 (13)
C4—C5—C6—C9	−174.94 (13)	C12—C13—C14—C9	0.6 (2)
N1—C5—C6—C9	3.7 (2)	C10—C9—C14—C13	1.4 (2)
C5—C6—C7—C8	1.2 (2)	C6—C9—C14—C13	179.09 (13)
C9—C6—C7—C8	176.70 (13)	C16—O7—C15—O8	2.6 (2)
C4—C3—C8—C7	1.2 (2)	C16—O7—C15—C12	−177.15 (13)
C2—C3—C8—C7	−177.57 (13)	C11—C12—C15—O8	178.12 (15)
C6—C7—C8—C3	−1.8 (2)	C13—C12—C15—O8	−2.7 (2)
C7—C6—C9—C14	−54.82 (18)	C11—C12—C15—O7	−2.1 (2)
C5—C6—C9—C14	120.00 (16)	C13—C12—C15—O7	177.07 (13)

### Hydrogen-bond geometry (Å, º)

**Table:**

*D*—H···*A*	*D*—H	H···*A*	*D*···*A*	*D*—H···*A*
C16—H16*A*···O3^i^	0.98	2.50	3.253 (2)	134
C1—H1*C*···O2^ii^	0.98	2.57	3.5214 (19)	164
C14—H14*A*···O2^iii^	0.95	2.47	3.2399 (19)	138
C8—H8*A*···O3^iv^	0.95	2.41	3.3239 (19)	162

Symmetry codes: (i) −*x*+2, −*y*+1, −*z*+1; (ii) −*x*, −*y*, −*z*+2; (iii) −*x*, −*y*, −*z*+1; (iv) *x*−1, *y*, *z*.
